# Comparison of the Blood–Brain Barrier Penetration Ability and Anti-Neuroinflammatory Activity of Chromones in Two Types of Agarwood

**DOI:** 10.3390/ph18040510

**Published:** 2025-03-31

**Authors:** Mengyuan Yang, Yanan Yuan, Jingfan Wei, Yifei Pei, Yuanfei Niu, Yifan Zhao, Xiangying Kong, Zhijie Zhang

**Affiliations:** 1 Institute of Chinese Materia Medica, China Academy of Chinese Medical Sciences, Beijing 100700, China; yyy250224@126.com (M.Y.); yfpei@icmm.ac.cn (Y.P.); n_yuanfei@163.com (Y.N.); yfzhao@icmm.ac.cn (Y.Z.); xykong@icmm.ac.cn (X.K.); 2 National Resource Center for Chinese Materia Medica, China Academy of Chinese Medical Sciences, Beijing 100700, China; ynyuan@icmm.ac.cn; 3 School of Polymer Science and Polymer Engineering, The University of Akron, Akron, OH 44325, USA; jw357@uakron.edu

**Keywords:** ordinary agarwood, Qi-Nan agarwood, blood–brain partitioning, neuroinflammation, chromones

## Abstract

**Background/Objectives**: Agarwood has a good neuroprotective effect and is often used to relieve anxiety and treat insomnia. This study compared the similarities and differences in the chromone components of two types of agarwood. It further investigated the absorption and brain distribution characteristics of these components in rats and their neuroprotective effects mediated through anti-neuroinflammatory pathways. **Methods**: This study confirmed, through ITS2 barcoding and chloroplast genome analysis, that both the ordinary and Qi-Nan agarwood are derived from *Aquilaria sinensis*. A comparative analysis of chromones in ethanol extracts derived from ordinary and Qi-Nan agarwood, as well as those capable of penetrating the blood-brain barrier in vivo, was conducted using UPLC-Q-TOF-MS. Subsequently, an in vitro neuroinflammatory model was established via lipopolysaccharide (LPS)-stimulated BV-2 cells to evaluate the anti-neuroinflammatory activity of differential chromones. **Results**: UPLC-Q-TOF-MS characterization revealed the chromone components in the two types of agarwood: A total of 81 chromone compounds were identified in the ethanol extracts of ordinary agarwood (OAE) (20 THPECs, 42 FTPECs, and 19 BI), while 41 were identified in the ethanol extracts of Qi-Nan agarwood (QNE) (11 THPECs and 30 FTPECs). Pharmacokinetic analysis in rats showed that 14 components from OAE (eight THPECs and six FTPECs) penetrated the rat serum, and 10 of these 14 components penetrated the blood–brain barrier (BBB). Twelve FTPECs from QNE penetrated the rat serum, all of which penetrated the BBB. The total peak area of the total ion current (TIC) was calculated for the samples, and the TIC of the serum was compared to that of the brain tissue from the same rat to roughly estimate the ratio. The results demonstrated that the capability of FTPECs to traverse the blood–brain barrier is substantially superior to that of THPECs. Correspondingly, only FTPECs were detected using DESI-MS imaging; no THPECs were detected in rat brain tissue, and DESI-MS imaging localized FTPECs to neuroanatomic regions (cerebral cortex, thalamus, and hippocampus). In vitro neuroinflammatory assays revealed the superior anti-inflammatory efficacy of QNE over OAE (IL-6/TNF-α suppression, *p* < 0.05), correlating with its FTPEC-rich composition. **Conclusions**: Structure–activity relationships identified FTPECs as potent inhibitors of pro-inflammatory cytokines, exhibiting enhanced BBB penetration (blood–brain relative abundance > 1). These findings establish FTPECs as prioritized candidates for CNS-targeted therapeutics, with QNE’s pharmacological superiority attributed to its FTPEC dominance and optimized BBB transit capacity.

## 1. Introduction

Agarwood, also known as eaglewood, gaharu, jinko, aloeswood, pokok karas, chenxiang, and kalambak, is highly valued for its distinctive and pleasing fragrance [1]. It is widely utilized in perfumes, aromatherapy, and traditional medicine [2]. As a valuable traditional Chinese medicine, agarwood exhibits distinct sedative, analgesic, and sleep aid properties and can be employed in the treatment of various diseases. Agarwood is primarily composed of resin and heartwood [3]. Resin, the primary component of agarwood, is secreted as a self-defense mechanism when the agarwood tree sustains an external injury.

Agarwood is classified into ordinary agarwood and Qi-Nan agarwood based on their distinctive aromas. Ordinary and Qi-Nan agarwood are utilized in the same clinical fields. However, there are notable differences in their aromas and compositions. In comparative studies of ordinary and Qi-Nan agarwood, Masakazu Ishihara [4] discovered in 1993 that the levels of 2-(2-phenylethyl)chromone and 2-[2-(4-methoxyphenyl)ethyl]chromone in Kanankoh agarwood are significantly higher than those in Jinko agarwood. Subsequent research has confirmed that the high concentrations of 2-[2-(4-methoxyphenyl)ethyl]chromone and 2-(2-phenylethyl)chromone are the key characteristics that define Qi-Nan agarwood [1,5,6].

**Figure 1:**
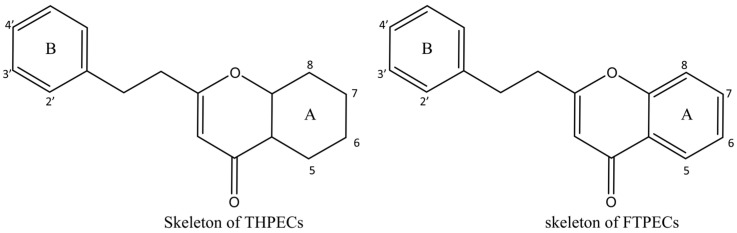
Skeleton structure of THPECs and FTPECs.

Both 2-[2-(4-methoxyphenyl)ethyl]chromone and 2-(2-phenylethyl)chromone belong to the 2-(2-phenylethyl)chromone (PEC) group. PECs are a type of compound with a phenylethyl substituent at the C2 position of the chromone (benzo-γ-pyrone) [7]. PECs can be categorized into five types based on the structural characteristics of the chromone skeleton: FTPECs (Flindersia-type 2-(2-phenylethyl)chromones), THPECs (5,6,7,8-tetrahydro-2-(2-phenylethyl)chromones), EPECs (mono-epoxy-5,6,7,8-tetrahydro-2-(2-phenylethyl)chromones), DEPECs (diepoxy-5,6,7,8-tetrahydro-2-(2-phenylethyl)chromones), and polymeric 2-(2-phenylethyl)chromones [8]. Our research group [5,9] identified 75 PECs in two types of agarwood, including 46 FTPECs, 15 THPECs, and four mono- and di-epoxy PECs, as well as 12 dimeric and trimeric chromones. The primary chromone types in agarwood are FTPECs and THPECs. The key distinction between THPECs and FTPECs lies in the saturation of the A ring [10]. THPECs have a highly oxidized cyclohexane ring in the A ring, whereas FTPECs have a benzene ring in the A ring (Figure 1). Qi-Nan agarwood contains significantly more FTPECs than ordinary agarwood. In contrast, ordinary agarwood contains significantly more THPECs than Qi-Nan agarwood. Therefore, these two types of chromones can serve as markers to differentiate ordinary agarwood from Qi-Nan agarwood. The majority of polymeric chromones in agarwood are dimers (BI) composed of two PEC monomers.

However, these chromone components are only found in a few plant species in nature, such as *Eremophila georgei* (Myoporaceae), *Bothriochloa ischaemum* (Gramineae), *Imperata cylindrica* (Gramineae), *Cucumis melo* L. (Cucurbitaceae), and *Aquilaria* spp. (Thymelaeaceae). In Thymelaeaceae species other than agarwood, the chromone concentration remains relatively low and is not considered a principal component. In contrast, chromones are the predominant component in agarwood [7,10].

Modern pharmacological research has demonstrated that chromone components have a significant protective effect on neuronal cells and exhibit inhibitory activity against acetylcholinesterase (AChE) [7]. Lin Yang [11] discovered that the compound 6,8-dihydroxy-2-[2-(3-hydroxy-4-methoxyphenyl)ethyl]chromone significantly inhibits the neurotoxicity of PC12 cells induced by glutamate and the neurotoxicity of human glioma U251 cells induced by corticosterone. Jeong Seon Yoon [12] found that the compounds 5-hydroxy-2-(2-phenylethyl)chromone and 5-hydroxy-2-[2-(2-hydroxyphenyl)ethyl]chromone demonstrated significant neuroprotective activity against glutamate-induced neurotoxicity in primary cultured rat cortical cells.

For a chemical component to exert its effect on an organism, it must penetrate both the body and, in some cases, the brain. The blood–brain barrier serves as the “gatekeeper” of the brain, shielding it from harmful substances such as pathogens. However, it also obstructs the entry of most drugs, leading to poor treatment outcomes for many neurological and metabolic diseases.

Numerous studies have demonstrated agarwood’s significant neuroprotective effects, with the chromones in agarwood identified as potential bioactive components. However, research on whether these chromones can reach brain tissue via blood circulation and the material basis for the neuroprotective effects of agarwood remains limited. Ordinary and Qi-Nan agarwood, two varieties of the same species, show distinct differences in different structural types of chromones. To compare their efficacy and explore variations in active chromones, this study used LPS-stimulated BV-2 cells to establish a neuroinflammatory model. This model assessed the anti-inflammatory effects of different active components, aiming to identify neuroprotective elements in two types of agarwood.

In this experiment, it was discovered that FTPECs possess a very strong ability to penetrate the blood–brain barrier. The inhibitory effects of the two types of agarwood and the components of FTPECs and THPECs on LPS-induced inflammation were compared, as well as the ability of the chromone components to penetrate the blood–brain barrier. A preliminary investigation was conducted into the relationship between the chromone compound’s structure and its ability to penetrate the blood–brain barrier.

## 2. Results

### 2.1. Species Identification

**Figure 2:**
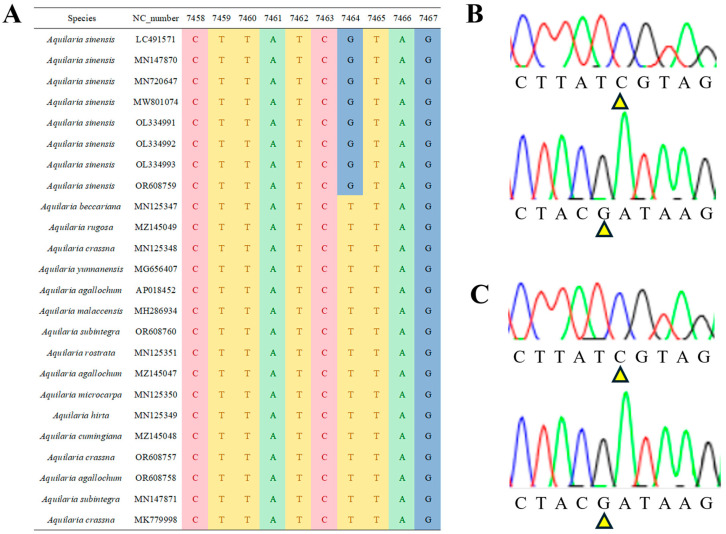
Species identification results using the Herb-Q method: 10 bp base information before and after the 7464 locus in the chloroplast genome of *Aquilaria* Species (**A**). The forward and reverse Sanger sequencing results of Sample A01 showed the expected results at the 7464 site (**B**). The forward and reverse Sanger sequencing results of Sample B01 showed the expected results at the 7464 site (**C**). Different colors of peaks correspond to different bases below. Red represents base T; green represents base A; black represents base G; blue represents base C.

Based on collection area information and morphological identification, Professor Zhijie Zhang from the Institute of Chinese Materia Medica, China Academy of Chinese Medical Sciences, and Shan Sun, president of the Beijing Agarwood Association, preliminarily identified the two collected plants as belonging to the *Aquilaria sinensis* species. Furthermore, ITS2 universal barcode sequencing and the Herb-Q [13,14,15] method were used for species identification. After aligning the chloroplast genomes, Cp7464 was identified as a specific SNP site for *Aquilaria sinensis* (Figure 2), which showed a G genotype for *Aquilaria sinensis*, while all other species of the genus *Aquilaria* showed a T genotype. Sanger sequencing of the A01 and B01 samples confirmed the expected genotype at this site (Figure 2). These results indicate that the two samples from different regions belonged to *Aquilaria sinensis*.

### 2.2. Comparative Analysis of the Chromones in Ordinary and Qi-Nan Agarwood

**Figure 3:**
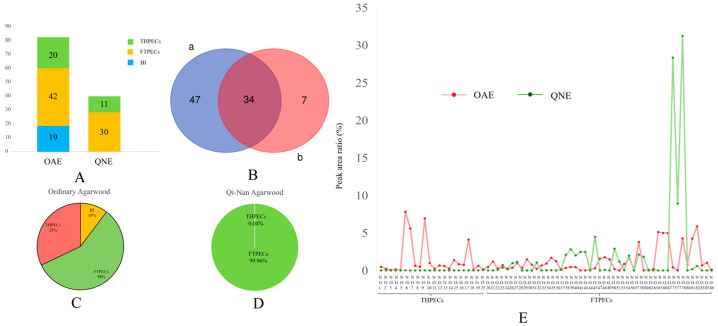
Results of the chromone components in ethanol extracts of two types of agarwood. The number of different chromone types in the ethanol extracts of two types of agarwood (**A**). Venn diagram of the chromone components in OAE (a) and QNE (b) (**B**). The relative peak area ratio of different types of chromones in the ethanol extracts of ordinary agarwood (OAE) (**C**). The relative peak area ratio of different types of chromones in the ethanol extracts of Qi-Nan agarwood (QNE) (**D**). Proportion of the relative peak area of each component of two types of agarwood (**E**).

Employing the UNIFI workstation, along with a literature review and comparison with reference substances, we compared and analyzed the retention times, parent ion mass-to-charge ratios, and fragment ions of the detected compounds. A total of 88 chromone components were identified through UPLC-Q-TOF-MS analysis of OAE and QNE, comprising 22 THPECs, 47 FTPECs, and 19 chromone dimers (BI). In total, 81 chromone components were identified in OAE, including 20 THPECs, 42 FTPECs, and 19 chromone dimers (BI), and 41 chromone components were identified in QNE, including 11 THPECs and 30 FTPECs (Figure 3A,B) (see Supplementary Materials Table S1).

Because the skeletons of the chromones are the same, the peak area and peak intensity of the compounds can roughly represent their content proportion. Therefore, the peak area of the mass spectrum peak of each identified compound was extracted, and the mass spectrum peak area ratio of each component was calculated. The chromone constituents of OAE were primarily composed of THPECs and FTPECs. In QNE, THPECs were detected in small quantities, whereas FTPECs were found at significantly higher concentrations. This indicates that THPECs were predominantly present in OAE, whereas FTPECs were the primary chromone components in QNE (Figure 3C–E).

### 2.3. Comparative Analysis of the Blood-Penetrating Chromones in the Two Types of Agarwood

UPLC-MS detection and analysis identified a total of 22 components in the drug-containing serum from the two groups of rats (Table 1). The serum from the ethanol extract of ordinary agarwood (OAS) contained 14 prototype chromones, including eight THPECs and six FTPECs, while the serum from Qi-Nan agarwood (QNS) contained 12 prototype chromones, all of which were FTPECs. This shows that both THPECs and FTPECs can penetrate the blood.

**Figure 4:**
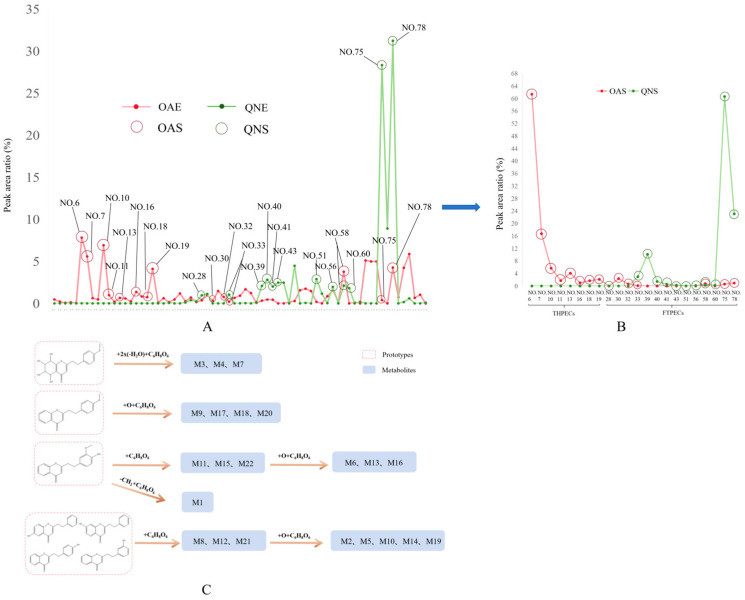
Results of the blood-penetrating components in two types of agarwood. The relative peak area ratio of each detected blood-penetrating component in two types of agarwood ethanol extracts and two types of agarwood serum: OAE and QNE (**A**) and OAS and QNS (**B**), respectively. The production pathway of each metabolite (**C**).

Further analysis indicated that THPECs with a high peak area ratio in OAE were more readily absorbed into the body. The eight blood-penetrating THPECs identified in OAS exhibited relatively high concentrations in OAE (Figure 4A), which likely explains their detectability in the blood. Of the six blood-penetrating FTPECs identified in OAS, two components were present at relatively high concentrations in OAE. The other four components did not exhibit a significant content advantage in OAE. However, these four components were still detected in the blood. Therefore, the detection of FTPECs in blood is related to their contents and structures.

Structural analysis of the chromone components absorbed from both types of agarwood revealed that many of these compounds contain –OH or –OCH_3_ substituents. These substituents are susceptible to metabolic transformations, including demethylation, hydroxylation, dehydration, and glucuronidation, which increase their likelihood of absorption.

For example, 2-[2-(3-methoxy-4-hydroxyphenyl)ethyl] chromone derivatives undergo phase I demethylation followed by phase II glucuronidation to form metabolite M1. 2-[2-(3-methoxy-4-hydroxyphenyl)ethyl]chromone class compounds undergo glucuronidation reactions to produce metabolites M11, M15, and M22. Additionally, these compounds undergo hydroxylation and glucuronidation to produce metabolites M6, M13, and M16. 6-hydroxy-2-(2-phenylethyl)chromone class compounds undergo hydroxylation and glucuronidation reactions to produce metabolites M2, M5, M10, M14, and M19. 7-hydroxy-2-(2-phenylethyl)chromone class compounds undergo glucuronidation reactions to produce metabolites M8, M12, and M21. 4′-methoxyagarotetrol class compounds first undergo dehydration reactions to lose two molecules of water and then undergo glucuronidation reactions to produce metabolites M3, M4, and M7. 2-[2-(4′-methoxyphenyl)ethyl]chromone class compounds undergo hydroxylation reactions and glucuronidation reactions to produce metabolites M9, M17, M18, and M20 (Figure 4) (see Supplementary Materials Table S2.)

### 2.4. Comparative Analysis of the Brain-Penetrating Chromone Components in the Two Types of Agarwood

A total of 21 brain-penetrating components were identified in the brain tissue of the two groups of rats, all of which were also present in the corresponding medicinal materials (Table 2). Brain tissue from the ordinary agarwood ethanol extract (OAB) contained 13 chromones, including five THPECs and eight FTPECs, while the Qi-Nan agarwood ethanol extract (QNB) contained 15 chromones, all FTPECs. The analysis revealed that both the THPECs and FTPECs contained compounds capable of crossing the blood–brain barrier.

**Figure 5:**
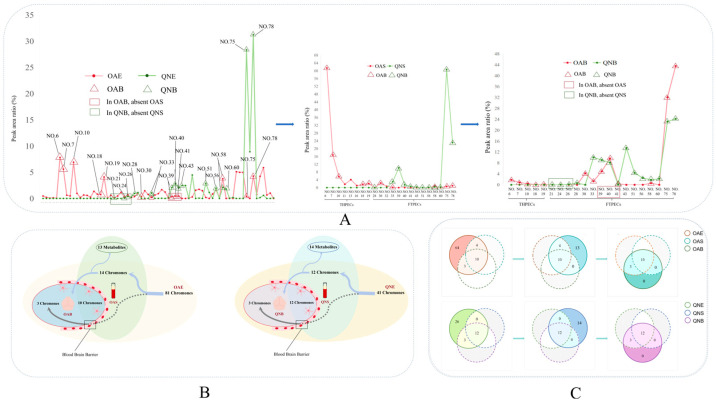
The results of the blood- and brain-penetrating components of two types of agarwood. Relative peak area ratio of the brain-penetrating components of two types of agarwood in ethanol extract, serum, and brain tissue (**A**). Transfer process of the brain-penetrating components of two types of agarwood in ethanol extract, serum, and brain tissue (**B**). The number of chromone components in the ethanol extract, serum, and brain tissue of two types of agarwood (**C**).

Five brain-penetrating THPECs were identified in OAB. These components were all detected in OAS and exhibited relatively high peak area ratios in OAE (Figure 5A). Structural analysis revealed that all these components possess substituent groups, with a relatively high number of –OH groups. This suggests that chromone components with –OH groups are more likely to penetrate the brain when present in sufficient amounts.

Eight brain-penetrating FTPECs were identified in OAB. Five of these components could be detected in OAS. However, the remaining components—2-[2-(4-hydroxyphenyl)ethyl]chromone (No. 39), 2-[2-(3-methoxy-4-hydroxyphenyl)ethyl]chromone (No. 40), and 7-hydroxy-2-(2-phenylethyl)chromone (No. 41)—were detected only in OAB, not in OAS, and exhibited low peak area ratios in OAE.

The QNB group had 15 brain-penetrating chromone components, all of which were FTPECs. Twelve of these components were also detected in QNS (Figure 5A). The Qi-Nan agarwood group had three components detected exclusively in QNB and not in QNS. These included 6-hydroxy-2-[2-(4-hydroxyphenyl)ethyl]chromone (No. 21), 6-hydroxy-2-[2-(4-hydroxyphenyl)ethyl]chromone (isomer 1) (No. 24), and 6-hydroxy-2-[2-(3-hydroxy-4-methoxylphenyl)ethyl]chromone (No. 26). These three components exhibited relatively low contents in QNE (Figure 5A).

Components No. 39, No. 40, and No. 41 were identified in the ethanol extracts of the two types of agarwood. These three components exhibited higher concentration ratios in QNE compared to OAE. Although absent in OAS, these components were detected in QNS, suggesting their ability to enter the body. These three components were identified in both OAB and QNB, suggesting that despite their low concentration ratio in the ordinary agarwood group, they accumulate in the brain after entering the body, facilitating their detection in brain tissue. Similarly, the three components detected exclusively in QNB and absent in QNS exhibited very low contents in QNE, further suggesting their accumulation in the brain after entering the body, which facilitates their detection in brain tissue.

### 2.5. The Transfer Process of the Chromone Components of the Two Types of Agarwood in Ethanol Extract, Serum, and Brain Tissue

In the OAE, a total of 14 prototype components were detected in OAS, and several metabolic transformations occurred in the body, resulting in the formation of 13 metabolites. Among these 14 prototype components, 10 were able to cross the blood–brain barrier via systemic circulation and enter rat brain tissue (OAB). Notably, three components, due to their low concentrations, were not detected in the serum. However, these three components were still able to enter the body, traverse the blood–brain barrier via systemic circulation, and accumulate in brain tissue.

Similarly, in the QNE, a total of 12 prototype components were detected in rat serum (QNS), and several metabolic transformations occurred in the body, resulting in the formation of 14 metabolites. All 12 prototype components were able to cross the blood–brain barrier via systemic circulation and enter rat brain tissue (QNB). Similarly, three components in Qi-Nan agarwood were not detected in the serum due to low content, but these three components passed through the blood–brain barrier via systemic circulation and accumulated in brain tissue (Figure 5B). Finally, 81, 27, and 13 chromone components were identified in the ethanol extract, drug-containing serum, and brain tissue of ordinary agarwood. Additionally, 41, 26, and 15 chromone components were identified in the ethanol extract, drug-containing serum, and brain tissue of Qi-Nan agarwood (Figure 5C).

### 2.6. Blood–Brain Relative Abundance of the Two Types of Chromones

Blood–brain partitioning (B/B) reflects a compound’s ability to penetrate the blood–brain barrier, calculated as the ratio of its concentration in brain tissue to that in blood. In this study, UPLC-MS was employed to detect chromone components from ethanol extracts of agarwood in both serum and brain tissues. The response value of the UPLC-MS results is related to the structure. Whether a compound is in the blood or brain, its response in mass spectrometry is similar. Because the skeletons of chromones are basically the same, the relative content of each compound can be roughly characterized by extracting the peak area of each compound in the mass spectrum. That is, by calculating the ratio of the peak area in brain tissue to the peak area in serum (blood–brain relative abundance of chromones), the ability of chromones to penetrate the blood–brain barrier (BBB) can be preliminarily evaluated.

**Figure 6:**
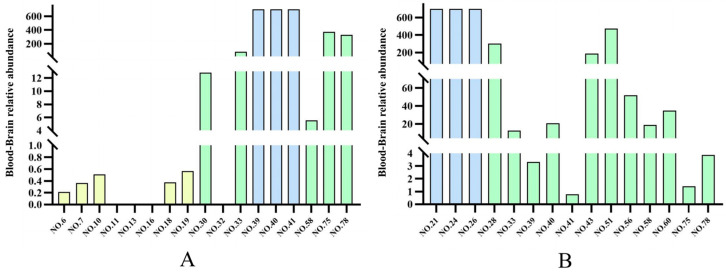
Blood–brain relative abundance of each component in two types of agarwood: ordinary agarwood (**A**) and Qi-Nan agarwood (**B**). Yellow represents THPECs; Green represents FTPECs; Blue represents the FTPECs with the highest blood–brain relative abundance.

Our experimental results demonstrate that both THPECs and FTPECs exhibited BBB permeability. Moreover, the ability of FTPECs to penetrate the BBB was significantly greater than THPECs. In OAS, the proportion of the peak area of THPECs was higher than that of FTPECs (Figure 5A). However, in OAB, the proportion of the peak area of THPECs was lower than that of FTPECs (Figure 5A). The calculation of the blood–brain relative abundance of chromones for ordinary agarwood components revealed striking differences: THPECs consistently showed a blood–brain relative abundance < 1, indicating limited brain penetration, while FTPECs demonstrated a significantly higher blood–brain relative abundance > 1. There were 15 brain-penetrating components in Qi-Nan agarwood, all of which were FTPECs. Only one of them had a blood–brain relative abundance less than 1, and the other components had a blood–brain relative abundance far greater than 1. These findings confirm that FTPECs possess superior BBB transit capability compared to THPECs, as evidenced by their order-of-magnitude greater blood–brain relative abundance (Figure 6).

### 2.7. Distribution of the Chromone Components in Brain Tissue

DESI-MS is employed to analyze the molecular spatial distribution characteristics of a sample by scanning the sample surface with mass spectrometry. DESI-MS exhibits lower detection sensitivity compared to UPLC-MS [16,17]. However, it enables direct observation of each group’s spatial distribution of brain-penetrating components in brain tissues.

**Figure 7:**
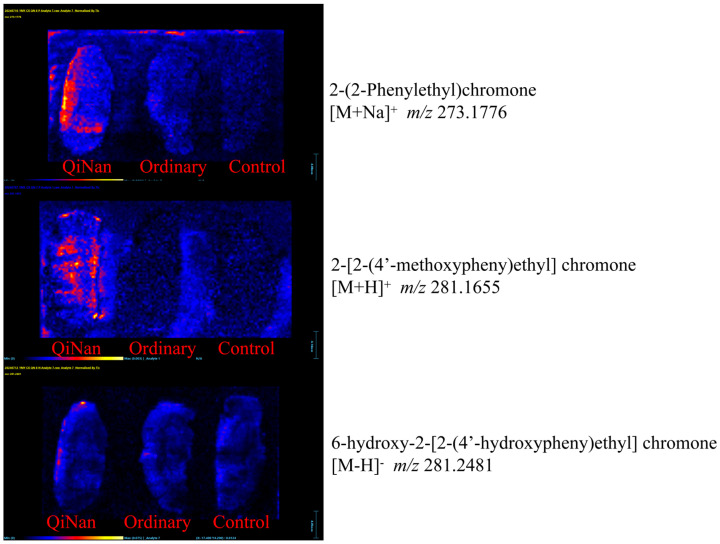
The distribution of the brain components in each group detected using DESI-MS.

In this study, frozen brain tissue slices from three rat groups (blank group, OAB, and QNB) were analyzed using DESI-MSI. Under the same administration conditions, DESI-MSI, despite its relatively low detection sensitivity, did not detect any brain-penetrating components in OAB, whereas several components were clearly observed in the brain tissue distribution of QNB. For instance, 2-(2-phenylethyl)chromone and 6-hydroxy-2-[2-(4′-hydroxyphenyl)ethyl]chromone were predominantly localized in the cerebral cortex, whereas 2-[(4′-methoxy)phenylethyl]chromone was detected across multiple brain regions, primarily in the thalamus and hippocampus, with minor distribution in the cerebral cortex (Figure 7).

Based on our aforementioned research, certain chromone components found in agarwood exhibit an enrichment phenomenon in the brain. Consequently, in our study, an in vitro neuroinflammation model was established using LPS (lipopolysaccharide) to investigate whether chromone components possess anti-neuroinflammatory pharmacological effects.

### 2.8. Comparison of the Anti-Neuroinflammation Effects of the Two Types of Agarwood and Their FTPEC and THPEC Components

**Figure 8:**
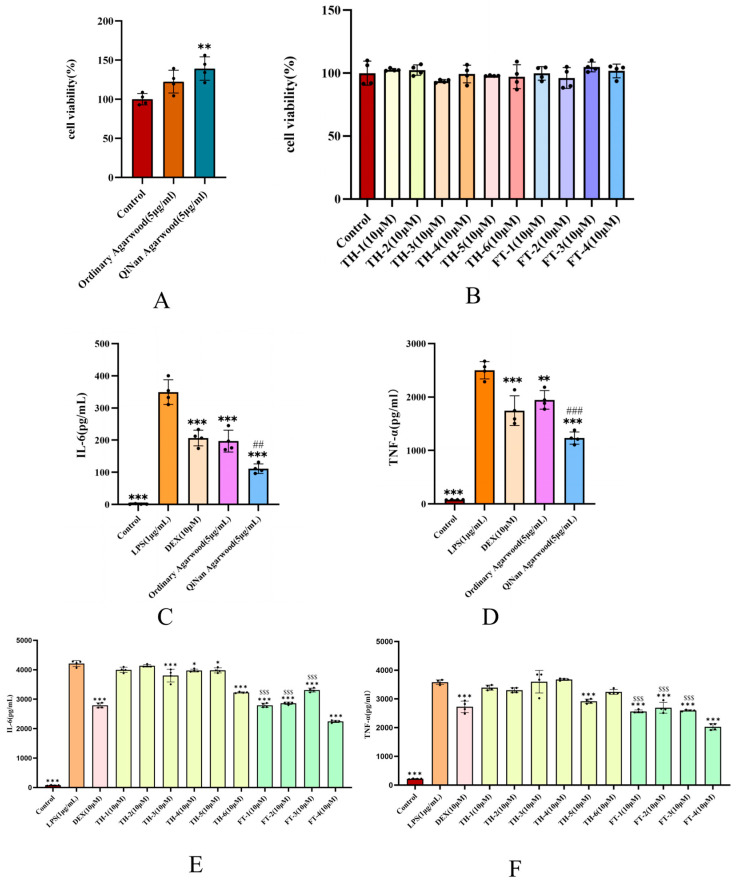
Cell experiment results. The CCK-8 results of BV-2 cells treated with ordinary agarwood (5 μg/mL) and Qi-Nan agarwood (5 μg/mL) (**A**). The CCK-8 results of BV-2 cells based on ten types of compounds (10 μM each) (**B**). Inhibitory effects of dexamethasone (Dex, 10 μM), ordinary agarwood (5 μg/mL), and Qi-Nan agarwood (5 μg/mL) on LPS-induced IL-6 production in BV-2 cells (**C**). Inhibitory effects of dexamethasone (Dex, 10 μM), ordinary agarwood (5 μg/mL), and Qi-Nan agarwood (5 μg/mL) on TNF-α production in BV-2 cells induced by LPS (**D**). Inhibitory effects of dexamethasone (Dex, 10 μM) and 10 compounds (10 μM) on LPS-induced IL-6 production in BV-2 cells (**E**). Inhibitory effects of dexamethasone (10 μM) and 10 compounds (10 μM) on TNF-α production in BV-2 cells induced by LPS (**F**). * Compared to the model group LPS, there are significant differences between the administration group and the model group, * *p* < 0.05, ** *p* < 0.002, and *** *p* < 0.001; # significant differences between the Qi-nan agarwood (5 μg/mL) group and the ordinary agarwood (5 μg/mL) group, ## *p* < 0.002, and ### *p* < 0.001; $ among the four FTPEC components, compared to compound FT-4, the tree compounds are significantly different from the FT-4 components, namely, FT-1, FT-2, and FT-3, and $$$ *p* < 0.001.

This experiment utilized LPS (1 μg/mL) to induce stimulation of the BV-2 cells, thereby establishing an in vitro neuroinflammation model. To determine whether the inhibitory effect in each group was associated with a reduction in cell number, a CCK-8 assay was employed to evaluate the impact of each group’s drug solution on cell proliferation. None of the drug groups exhibited significant cytotoxicity in the BV-2 cells after 24 h of treatment (Figure 8A,B).

Compared to the model group LPS, dexamethasone and ethanol extracts of the two types of agarwood all inhibited the release of inflammatory factors IL-6 and TNF-α induced by LPS-stimulated BV-2 cells. The inhibitory effect of 5 μg/mL of OAE was comparable to that of dexamethasone (Dex, 10 μM). The inhibitory effect of 5 μg/mL of QNE was significantly greater than that of 5 μg/mL of OAE (*p* < 0.05) (Figure 8C,D).

At the same treatment concentration, compared to the THPECs, the FTPECs exhibited the strongest inhibitory effect on IL-6 expression in the BV-2 cells stimulated by LPS. Compared to the model group LPS, dexamethasone and all four FTPECs effectively inhibited the release of the inflammatory factor IL-6 in the BV-2 cells stimulated by LPS (Figure 8E). Among the six THPECs, four exhibited inhibitory effects on the release of inflammatory factor IL-6 in the BV-2 cells stimulated by LPS, including TH-3, TH-4, TH-5, and TH-6. Additionally, TH-3 and TH-6 demonstrated more pronounced inhibitory effects. Among the four FTPECs, FT-4 exhibited the strongest inhibitory effects on the production of IL-6 induced by LPS in the BV-2 cells. The inhibitory effects of the other three FTPECs were significantly weaker than that of FT-4.

At the same treatment concentration, compared to the THPECs, the FTPECs exhibited the strongest inhibitory effect on TNF-α expression in the LPS-stimulated BV-2 cells. Compared to the model group LPS, dexamethasone and four FTPECs inhibited LPS-induced TNF-α release in the BV-2 cells. Among the six THPECs, only TH-5 showed inhibitory activity. FT-4 exhibited the strongest inhibition among the FTPECs, with the other tree showing significantly weaker effects (Figure 8F).

The experiments demonstrated that the two types of agarwood have anti-inflammatory effects. QNE was significantly better than OAE in inhibiting inflammatory factors. Only four of the six THPEC components had anti-inflammatory effects, while all four FTPECs had anti-inflammatory effects, and the anti-inflammatory effects of the FTPECs were better than THPECs. Moreover, the results in Section 2.2 indicate that UPLC-Q-TOF-MS identified chromone components in OAE and QNE, revealing that OAE contained THPECs, FTPECs, and BI components, whereas QNE primarily consisted of FTPECs. Thus, it is hypothesized that the abundance of FTPECs in QNE may contribute to its superior inhibitory effect.

## 3. Discussion

In Section 2.1 (Species Identification) and as reported in the literature [18], DNA barcode analysis confirmed that both ordinary and Qi-Nan agarwood are derived from *Aquilaria sinensis*. According to the situation understood from visiting the growers of Qi-Nan agarwood, it is speculated that natural Qi-Nan agarwood should be a phenotypic variation in ordinary agarwood. However, significant compositional differences exist between the two types of agarwood despite originating from the same species [2,5]. The formation of agarwood is the resin produced by plants of the Thymelaeaceae family after being injured by the outside world [19]. Under environmental stress, plants produce substantial secondary metabolites as a self-protective mechanism [20]. Chromones are key secondary metabolites generated by agarwood in response to injury [21]. Type III polyketide synthases (PKSs) are involved in the biosynthesis of plant secondary metabolites, including polyketides [22]. Mengjun Xiao identified three highly expressed type III PKSs in agarwood [23]. Therefore, it is inferred that the synthesis of 2-(2-phenylethyl)chromones (PECs) in agarwood is related to PKSs. However, when comparing the differences in PECs between ordinary and Qi-Nan agarwood, it was found that ordinary agarwood has a more diverse range of PECs, including THPECs, FTPECs, and PECs dimers (BI). In contrast, Qi-Nan agarwood mainly contained FTPECs, with extremely low levels of THPECs (accounting for only 0.04% of the peak area), and no BI components were detected. It is speculated that this experimental result may be related to the different expression levels of PKSs in the two types of agarwood. Compared to ordinary agarwood, Peiwen Sun [24] identified 12 PKSs with higher expression levels in Qi-Nan agarwood, which are associated with injury-induced responses. Under environmental stress, these genes exhibit higher upregulation in Qi-Nan agarwood compared to ordinary agarwood. The genes involved in the biosynthesis of phenylpropanoids and flavonoids are more highly expressed in Qi-Nan agarwood than in ordinary agarwood. However, Peiwen Sun identified one PKS with high expression in ordinary agarwood. Integrating the synthetic pathway of 2-(2-phenylethyl)chromones (PECs) [25], Wei Li [26] proposed that DEPECs serve as precursors, EPECs and THPECs act as intermediate products, and FTPECs are the final products. Therefore, it is speculated that the high expression of PKSs in Qi-Nan agarwood promotes the synthesis of the final product (FTPECs) in 2-(2-phenylethyl)chromones (PECs). In ordinary agarwood, the expression level of PKSs is lower than that in Qi-Nan agarwood; hence, ordinary agarwood contains 2-(2-phenylethyl)chromones (PECs) from all stages.

The ability of central nervous system (CNS) drugs to cross the blood–brain barrier depends on several physicochemical properties, including lipophilicity, total polar surface area (TPSA), charge state, molecular size, flexibility, and hydrogen bond potential [27]. Arup Ghose [28] analyzed 317 CNS drugs and found that the optimal molecular weight range for CNS drugs is 300~350. Additionally, the logP values of most CNS drugs fall within the range of 2~4, suggesting that these drugs are predominantly lipophilic. In this study, 69 chromone monomer components were identified in the two types of agarwood, with molecular weights ranging from 250 to 397 Da. However, only 21 of these compounds were able to cross the blood–brain barrier. This suggests that molecular weight is not the primary determinant of brain penetration. Among the 21 compounds that enter the brain, three FTPECs from ordinary agarwood (No. 30, No. 33, and No. 75) were detected in both rat serum and brain tissue. However, these three compounds did not exhibit an advantageous peak area ratio in OAE, indicating that their content in the extract was low. This suggests that the content is not the primary factor influencing brain penetration and that the molecular structure may play a significant role.

There were three FTPEC components (No. 39, No. 40, and No. 41) in ordinary agarwood. Although it could be detected in OAB, it was not detected in OAS, and the content of these three components in OAE was low. It is speculated that these three components can easily enter the body, especially by penetrating the blood–brain barrier and forming an accumulation in brain tissue. Similarly, three FTPEC components were also detected in Qi-Nan agarwood (No. 21, No. 24, and No. 26). The content in QNE was low and was not detected in the QNS but was detected in QNB. These six components were all FTPECs, indicating that some components of FTPECs can easily pass through the blood–brain barrier and enter the nervous system. Additionally, this study identified that both THPECs and FTPECs are capable of passing through the blood–brain barrier. By comparing the blood–brain relative abundance of the blood and brain components of the two types of agarwood, it was found that the blood–brain relative abundance of FTPECs was higher than that of THPECs, indicating that components with FTPEC structures are more likely to enter the nervous system.

Lipid solubility is a crucial determinant of drug pharmacokinetics, influencing characteristics such as absorption, permeation, and elimination, and is closely related to lipophilicity [29]. The n-octanol/water partition coefficient (log Po/w) has been widely adopted as a fundamental physicochemical parameter in biomedical research and drug development [30]. Moreover, log Po/w is an essential indicator for evaluating the potential of compounds to penetrate the blood–brain barrier (BBB), with higher log Po/w values indicating greater lipophilicity, where a log Po/w of 2~4 indicates a higher likelihood of BBB penetration [30,31]. The topological polar surface area (TPSA), defined as the sum of the surface areas of molecular side blocks on non-hydrogen atoms around polar atoms in a molecule [32], also affects blood–brain barrier permeability. Generally, larger TPSA values hinder compound entry into the brain [33]. van de Waterbeemd reported that the TPSA of CNS drugs was typically less than 90 Å^2^ [34]. In this study, the log Po/w and TPSA of 21 brain-penetrating chromone components were estimated using the SwissADME prediction tool (http://www.swissadme.ch/ (accessed on 23 March 2025)) to reveal distinct physicochemical profiles between THPECs and FTPECs. The results revealed that for five THPECs, the log Po/w values ranged from 0.26 to 1.16, and the TPSA values ranged from 90.90 to 120.36 Å^2^. In contrast, for 16 FTPECs, the log Po/w values were between 2.76 and 3.73, and the TPSA values were between 30.21 and 79.90 Å^2^ (Table 3). The significant differences in physicochemical properties between FTPECs and THPECs indicate that FTPECs have greater potential for BBB penetration. This is supported by their advantageous profile of high lipophilicity and low polar surface area (TPSA < 90 Å^2^), which matches the recognized standards for compounds capable of penetrating the CNS [31,34].

In this study, using an in vitro neuroinflammation model, we demonstrated that both ordinary and Qi-Nan agarwood possess anti-neuroinflammatory effects. When the nervous system is pathologically injured, neuroglial cells, which function as immune cells in the nervous system, are activated, releasing a large number of pro-inflammatory factors. This immune response is termed neuroinflammation. The primary function of this response is to clear dead cells and restore brain function. However, the prolonged presence of these inflammatory factors in the brain can injure the blood–brain barrier, impair neurons, promote neuronal degeneration and death, and contribute to the development of various neurodegenerative diseases [35,36,37]. Studies have shown that neuronal apoptosis and neuroinflammation are significant risk factors for Alzheimer’s disease (AD) [35]. Therefore, agarwood has great potential for the treatment of neurodegenerative diseases. In the Chinese Pharmacopoeia, only ordinary agarwood from *Aquilaria sinensis* can be used as medicinal materials. Based on this study and previous research [5,9], we will carry out more experimental studies to recommend that the Chinese Pharmacopoeia update the original plant source of agarwood to include “*Aquilaria sinensis* and its cultivated varieties”, categorizing the medicinal materials as “agarwood” and “Qi-Nan agarwood”. Furthermore, the content determination and characteristic fingerprinting of Qi-Nan agarwood should be clearly distinguished from those of ordinary agarwood.

Meanwhile, to gain a deeper understanding of the pharmacological effects of different types of chromones (THPECs and FTPECs) in agarwood, this study used an in vitro neuroinflammation model and found that although both THPECs and FTPECs have anti-neuroinflammatory effects, FTPECs has a stronger anti-neuroinflammatory effect. Given the strong ability of FTPECs to pass through the blood–brain barrier and their anti-neuroinflammatory effects, FTPECs can be considered potential drugs for the treatment of neurological diseases.

## 4. Materials and Methods

### 4.1. Materials

#### 4.1.1. Samples and Reference Substances

Samples: Samples of ordinary and Qi-Nan agarwood were selected from 24 batches of samples (for details, see Supplementary Materials File S1). Species identification of the original plants for both types of agarwood samples was conducted using DNA barcoding techniques.

Reference Substances: Agarotetrol (Chengdu Push Bio-Technology Co., Ltd. (Chengdu, China), PS011165); 4′-methoxyagarotetrol (Chengdu Alfa Biotechnology Co., Ltd., (Chengdu, China), AFDB2201); isoagarotetrol (Chengdu Alfa Biotechnology Co., Ltd., (Chengdu, China), AFCF2105); 4′-methoxyisoagarotetrol (Chengdu Alfa Biotechnology Co., Ltd., (Chengdu, China), AFDI3001); 6,7-dihydroxy-2-(2-phenylethyl)-5,6,7,8-tetrahydrochromone (Chengdu Push Bio-Technology Co., Ltd., (Chengdu, China), PS013272); (5S,6S,7S,8R)-8-chloro-5,6,7-trihydroxy-2-(2-phenylethyl)-5,6,7,8-tetrahydrochromen-4-one (ChemFaces, (Wuhan, China), CFS202301); 2-(2-phenylethyl)chromone (Chengdu Push Bio-Technology Co., Ltd., (Chengdu, China), PS020524); 6-hydroxy-2-(2-phenylethyl)chromone (Chengdu Alfa Biotechnology Co., Ltd., (Chengdu, China), AFCB2104); 2-[2-(4′-methoxyphenyl)ethyl]chromone (Chinese Academy of Tropical Agricultural Sciences, (Haikou, China)); 6-methoxy-2-[2-(3′-methoxyphenyl)ethyl]chromone (Chengdu Alfa Biotechnology Co., Ltd., (Chengdu China), AFDI3002).

#### 4.1.2. Equipment and Reagents

Equipment: ACQUITYI-class UPLC (Waters, Milford, CT, USA); Xevo G2-XS Q TOF MS (Waters, Milford, CT, USA); DESI (Waters, Milford, CT, USA); Acquity UPLC^®^ HSST3 C18 chromatographic column (2.1 × 100 mm, 1.8 µm, Waters, Milford, CT, USA); FA1004-type thousandth analytical electronic balance (Shanghai Liangping Instrument Co., Ltd. (Shanghai, China)); AUW12OD-type ten-thousandth electronic balance (Shimadzu Corporation, Tokyo, Japan); SK7210HP-type ultrasonic instrument (Shanghai Kedao Ultrasonic Instruments Co., Ltd. (Shanghai, China)); N13224-type intelligent sample grinder (Beijing Hered Technology Co., Ltd. (Beijing, China)); ATCN-12-type nitrogen blowing instrument (Beijing Antai Ruike Technology Co., Ltd. (Beijing, China)); 101-1AB-type electric heat blast drying oven (Beijing Zhongxing Weiye Instrument Co., Ltd. (Beijing, China)); N-1100-type rotary evaporator (Shanghai Ailang Instrument Co., Ltd. (Shanghai, China)); freeze dryer: ALPHA 1-2 LD Plus (Osterode, Germany); high-speed refrigerated centrifuge 5922 (Kubota Corporation, Osaka, Japan); high-throughput tissue grinder SCIENTZ-48 (Ningbo Xinzhi Biotechnology Co., Ltd. (Ningbo, China)); CryoStar NX70 freezing microtome (Thermo Fisher Scientific, Waltham, MA, USA); NanoDrop 2000-type ultra-micro spectrophotometer (Thermo Fisher Scientific, Waltham, MA, USA); T100^TM^ instrument (Bio-Rad, Hercules, CA, USA); DYY-8C-type electrophoresis instrument (Beijing Liuyi Instrument Factory, (Beijing, China)); JY04S-3C type gel imager (Beijing Junyi Oriental Electrophoresis Equipment Co., Ltd., (Beijing, China)); PyroMark Q48-type pyrophosphate sequencing instrument (QIAGEN, Hilden, Germany).

**Reagents:** Methanol (HPLC grade, F24O3C201, Thermo Fisher Chemical, Waltham, MA, USA), acetonitrile (HPLC grade, F24O1N209, Thermo Fisher Chemical,Waltham, MA, USA), corn oil (Biosharp, (Hefei, China)), penicillin–streptomycin solution (Aladdin, P301861, (Shanghai, China)), DMSO (Solarbio, D8371, (Beijing, China)), DMEM (Gibco, 10566016, GrandIsland, New York, USA), FBS (Gibco, 10099141C, GrandIsland, New York, USA), trypsin EDTA solution A (VivaCell Biosciences. 2338301, (Shanghai, China)), dexamethasone (Shanghai Yuanye Bio-Technology Co., Ltd. (Shanghai, China) B25793-100mg), lipopolysaccharide (Sigma L2630-100mg, St Louis, Missouri, USA), Cell Counting Kit-8 (NCM, 20240801, (Suzhou, China)), Mouse IL-6 ELISA Kit (Solarbio, SEKM-0007, (Beijing, China)), Mouse TNF-α ELISA Kit (Solarbio, SEKM-0034, (Beijing, China)), high-efficiency plant genomic DNA extraction kit (Beijing Tiangen Biochemical Technology Co., Ltd., (Beijing, China), batch number DP320-03), and 2×Taq Master Mix (TaKaRa Company, Japan, batch number Rk20429).

#### 4.1.3. Cells and Animals

BV-2 cells were kindly provided by Cell Bank, Chinese Academy of Sciences, Shanghai, China.

Eight-week-old male Wistar rats of SPF grade, weighing 270 ± 10 g and purchased from Beijing Weitong Lihua Experimental Animal Technology Co., Ltd. (Beijing, China), were used in this study. The animal experimental ethics approval number is 2024B103. The rats were acclimated to the feeding conditions for 7 days at the Institute of Chinese Materia Medica, China Academy of Chinese Medical Sciences, under the license number SYXK (Jing) 2023-0077.

### 4.2. Methods

#### 4.2.1. Species Identification

Fresh leaves of ordinary and Qi-Nan agarwood were taken, designated as A01 and B01, respectively. DNA was extracted and amplified using universal primer ITS2 S2F (5’-ATGCGATACTTGGTGTGAAT-3’)/S3R (5’-GACGCTTCTCCAGACTACAAT-3’). The amplification products were then sequenced using the Sanger method, and the double-end sequencing results were aligned with the NCBI (https://www.ncbi.nlm.nih.gov/ (accessed on 20 October 2024.)) BLAST+ 2.16.0 database.

By searching the GenBank database in NCBI, 26 chloroplast genomes of 12 species of *Aquilaria* were obtained. After aligning and proofreading the sequences, we selected candidate SNP sites for *Aquilaria sinensis* detection using the Herb-Q method [13,14,15]. Specific PCR amplification primers Cp F (5’-GGGAATGATGAATCATAAAAAGAG-3’)/R(5’-GTTTTGGTCCCGCTATTCGA-3’) were designed using PyroMark ADSW 2.0 software. High-quality PCR products were obtained using the same amplification system and reaction conditions as for ITS2 S2F/S3R. The base types at the candidate SNP sites of samples A01 and B01 were determined by Sanger sequencing to further achieve accurate species identification.

#### 4.2.2. Preparation of Ethanol Extracts from the Two Types of Agarwood

Ethanol (95%) was used to extract ordinary and Qi-Nan agarwood, respectively, to prepare ethanol extracts of the two types of agarwood, which were then freeze-dried to prepare freeze-dried powder for later use.

#### 4.2.3. Preparation of the Administration Solution

An appropriate amount of the freeze-dried powder from the ethanol extracts of two types of agarwood was weighed, and an appropriate amount of corn oil was added to dissolve and suspend it. The drug administration solution was prepared at a dosage of 2 g/kg body weight of the crude drug, with a dosing volume of 1 mL per 100 g of rat body weight for administration to the rats.

#### 4.2.4. Animal Administration and Grouping

Thirty-six eight-week-old male Wistar rats were adapted to the feeding conditions for one week. Rats in the ordinary and Qi-Nan agarwood groups received an intragastric administration of the ethanol extract of ordinary or Qi-Nan agarwood at a dosage of 2 g/kg of crude drug, respectively. Rats in the blank group were intragastrically administered an equal volume of corn oil. This administration regimen was maintained for five days. Two hours after the final administration, the rats were anesthetized, and blood was collected from the abdominal aorta, while serum and brain tissue of the rats in each group were obtained and stored at −80 °C for further analysis.

#### 4.2.5. Serum and Brain Tissue Processing

The serum from 6 rats in each group was combined to make two mixed sera per group. The components in the serum were extracted with methanol. Four times, methanol was added into 500 μL of mixed serum, vortexed for 1 min, ultrasonicated in an ice bath for 15 min, and centrifuged (4 °C, 12,000 r/min, 10 min), and the supernatant was placed into a 2 mL Ep tube and blow dried under nitrogen gas. The residue was redissolved with 300 μL of methanol, vortexed for 1 min, ultrasonicated in an ice bath for 15 min, and centrifuged (4 °C, 12,000 r/min, 10 min), and the supernatant was stored in a 4 °C refrigerator for later use.

The brain tissue from 6 rats in each group was weighed, and an equal amount of pre-cooled physiological saline was added and ground into a homogenate using a tissue grinder. An appropriate amount of brain tissue homogenate was taken, and the components in the homogenate were extracted using methanol, which was the same as that in the drug-containing serum. Methanol extraction was repeated twice.

#### 4.2.6. Preparation of Brain Slices

Half of the brain tissue from each group of rats (6 rats) was used for the preparation of brain slices. The temperature of the freezing slice cabinet was set to −25 °C, and the blade temperature was set to −20 °C. The final thickness of the brain slices was 14 μm.

#### 4.2.7. UPLC-MS Analysis

UPLC-MS Conditions:

The liquid mass data acquisition used the UPLC-Q-TOF-MS system.

Chromatographic conditions: Waters Acquity UPLC^®^ HSST3 C18 chromatographic column (2.1 × 100 mm, 1.8 µm); mobile phase consisting of acetonitrile (A) and 0.1% formic acid water (B); flow rate: 0.3 mL/min; gradient elution program: 0–4.40 min, 5–18% A; 4.40–9.60 min, 18–25% A; 9.60–11.60 min, 25–35% A; 11.60–15.00 min, 35–40% A; 15.00–20.00 min, 40–52% A; 20.00–23.00 min, 52–85% A; 23.00–35.00 min, 85% A; column temperature, 30 °C; injection volume, 1 µL; UV scanning range, 190–500 nm.

Mass spectrometry conditions: Xevo G2-XS Q-TOF-MS system, ESI ion source, positive and negative ion modes. MS parameter settings: mass scanning range, 50–1500 Da; drying gas (N2) flow rate, 800 L/h; temperature, 450 °C; ion source temperature, 120 °C; cone gas flow rate, 50 L/h; cone voltage, 40 V; capillary voltage, +3.0 and −2.5 kV. In MSE mode, the low collision energy was 6 V, and the high collision energy was 15–50 V. Leucine Enkephalin solution (200 pg/μL) was used as an external standard solution for real-time mass calibration, with a flow rate of 5 μL/min, forming calibration ions, *m/z* 554.2615 [M − H]^−^ in negative ion mode, *m/z* 556.2771 [M + H]^+^ in positive ion mode.

UNIFI conditions: retention time range of 0~35.0 min; mass error of less than 10 ppm; positive ion mode adducts were +H, +Na, +K, and +NH_4_; negative ion mode adducts were –H, –HCOO, and –Cl.

#### 4.2.8. DESI-MS Analysis

DESI-MS conditions: DESI-MS was used to detect the spatial distribution of molecules on the surface of rat brain tissue slices. The DESI-MS conditions were a capillary voltage of +5.0 and −4.5 kV, a cone voltage of 40 V, and an ion source temperature of 150 °C. The acquisition mode was DESI-MS full scan, with a mass range of *m/z* 50–1200, the collision energy off, and the mass analyzer in sensitivity mode. The spatial resolution was 300 × 300 μm, and the scanning speed was 300 μm/s. The spray solvent was a 95% methanol–water solution containing 200 pg/μL of LE and 0.1% formic acid, with a spray rate of 5 μL/min, a nebulization gas N_2_ pressure of 0.5 Mpa, and a spray angle of 60°. The instrument signal strength was tuned to above 1e6 with a red Sharpie pen (rhodamine 6G, *m*/*z* 443).

Mass Spectrometry Imaging Data Processing: The frozen sections of rat brain tissue were scanned using MSI in both positive and negative ion modes. The data were imaged using HDImaging v1.4 software and compared to the liquid mass detection results to speculate on the compounds.

#### 4.2.9. Comparison of the In Vitro Activities of the Different Types of Chromones

Establishment of an in vitro neuroinflammation model: The BV-2 cells were suspended and inoculated into a 12-well culture plate (1.3 × 10^5^ cells/well) and cultured in an incubator set to 37 °C with 5% CO_2_ for 24 h. The positive drug group (10 μM of dexamethasone) and the treatment groups (5 μg/mL of OAE and QNE and 10 μM of 6 THPECs and 4 FTPECs) were added to a certain concentration of the corresponding drug solutions (Table 4). The model group (1 μg/mL of LPS) and the blank group were added to equal amounts of complete culture medium. After drug pretreatment for 2 h, a certain amount of LPS was added to both the model group and the treatment groups, and the blank group was added to an equal amount of complete culture medium. After continued culturing for 12 h, the cell culture fluid from each well was collected and centrifuged at 4 °C and 1000× *g* for 10 min, and the supernatant was collected. The concentrations of IL-6 and TNF-α in the supernatant were measured using ELISA kits.

CCK-8 Assay: The BV-2 cells were suspended and inoculated into a 96-well culture plate (5000 cells/well) and cultured in an incubator set to 37 °C with 5% CO_2_ for 24 h. The experiment included a blank cell group, a blank solvent group, and a drug group, with 4–6 replicate wells for each. After the addition of the corresponding drug treatments, the cultures were incubated for an additional 24 h. Then, 10 μL of CCK-8 solution was added to each well, and the plates were incubated for 1–4 h. Finally, the absorbance of each well was measured at 450 nm.

## 5. Conclusions

Both ordinary and Qi-Nan agarwood are derived from *Aquilaria sinensis.* Although there were great differences in the chromone components between the ordinary and Qi-Nan agarwood, the chromone components that can penetrate the blood–brain barrier in the two agarwood types were almost similar. That is, the two types of agarwood have similar active ingredients in the treatment of nervous system diseases.

Both ordinary and Qi-Nan agarwood hold promise for the treatment of neurodegenerative diseases, with chromones being the primary active components. Both THPECs and FTPECs can cross the blood–brain barrier to exert anti-neuroinflammatory effects. However, FTPECs demonstrate a stronger ability to penetrate the blood–brain barrier and a more potent anti-neuroinflammatory action. Therefore, both THPECs and FTPECs have the potential to be used in nervous system medicines, with FTPECs holding greater potential.

## Figures and Tables

**Table 1:** Blood-penetrating chromone components in two types of agarwood.

No.	Type	Component Name	OAS	QNS
6	THPECs	Agarotetrol	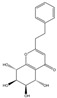	
7	THPECs	4′-methoxyagarotetrol	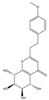	
10	THPECs	Agarotetrol (isomer 1)	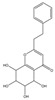	
11	THPECs	4′-methoxyagarotetrol (isomer 2)	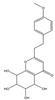	
13	THPECs	5,6,7-trihydroxy-5,6,7,8-tetrahydro-2-(2-phenylethyl)chromone	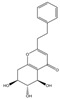	
16	THPECs	6,7-dihydroxy-5,6,7,8-tetrahydro-2-(2-phenylethyl)chromone	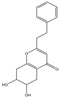	
18	THPECs	8-chloro-5,6,7-trihydroxy-2-(4-methoxyphenethyl)-5,6,7,8-tetrahydrochromone	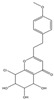	
19	THPECs	8-chloro-5,6,7-trihydroxy-2-(2-phenylethyl)-5,6,7,8-tetrahydrochromon	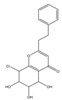	
28	FTPECs	6-hydroxy-2-[2-(3-methoxyl-4-hydroxyphenyl)ethyl]chromone		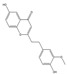
30	FTPECs	6-methoxy-2-[2-(3-hydroxy-4-methoxyphenyl)ethyl]chromone	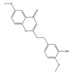	
32	FTPECs	6-methoxy-7-hydroxy-2-[2-(4-methoxyphenyl)ethyl]chromone	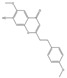	
33	FTPECs	2-[2-(3-hydroxyphenyl)ethyl]chromone	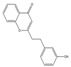	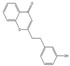
39	FTPECs	2-[2-(4-hydroxyphenyl)ethyl]chromone		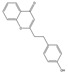
40	FTPECs	2-[2-(3-methoxy-4-hydroxyphenyl)ethyl]chromone		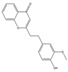
41	FTPECs	7-hydroxy-2-(2-phenylethyl)chromone		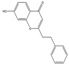
43	FTPECs	2-[2-(3-hydroxy-4-methoxyphenyl)ethyl]chromone		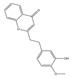
51	FTPECs	2-[2-(2-hydroxyphenyl)ethyl]chromone		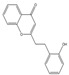
56	FTPECs	6-hydroxy-2-[2-(3-methoxyphenyl)ethyl]chromone		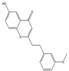
58	FTPECs	6-hydroxy-2-(2-phenylethyl)chromone	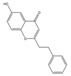	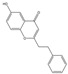
60	FTPECs	6-methoxy-2-[2-(3-methoxyphenyl)ethyl]chromone		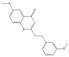
75	FTPECs	2-[2-(4-methoxyphenyl)ethyl]chromone	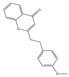	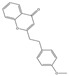
78	FTPECs	2-(2-phenylethyl)chromone	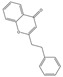	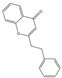

**Table 2:** Brain-penetrating chromone components in the two types of agarwood.

No.	Type	Component Name	OAB	QNB
6	THPECs	Agarotetrol	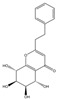	
7	THPECs	4′-methoxyagarotetrol	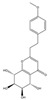	
10	THPECs	Agarotetrol (isomer 1)	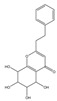	
18	THPECs	8-chloro-5,6,7-trihydroxy-2-(4-methoxyphenethyl)-5,6,7,8-tetrahydrochromene	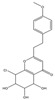	
19	THPECs	8-chloro-5,6,7-trihydroxy-2-(2-phenylethyl)-5,6,7,8-tetrahydrochromon	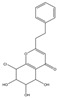	
21	FTPECs	6-hydroxy-2-[2-(4-hydroxyphenyl)ethyl]chromone		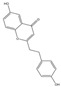
24	FTPECs	6-hydroxy-2-[2-(4-hydroxyphenyl)ethyl]chromone (isomer 1)		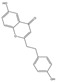
26	FTPECs	6-hydroxy-2-[2-(3-hydroxy-4-methoxylphenyl)ethyl]chromone		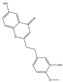
28	FTPECs	6-hydroxy-2-[2-(3-methoxyl-4-hydroxyphenyl)ethyl]chromone		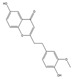
30	FTPECs	6-methoxy-2-[2-(3-hydroxy-4-methoxyphenyl)ethyl]chromone	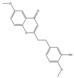	
33	FTPECs	2-[2-(3-hydroxyphenyl)ethyl]chromone	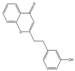	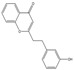
39	FTPECs	2-[2-(4-hydroxyphenyl)ethyl]chromone	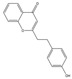	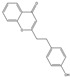
40	FTPECs	2-[2-(3-methoxy-4-hydroxyphenyl)ethyl]chromone	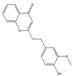	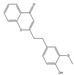
41	FTPECs	7-hydroxy-2-(2-phenylethyl)chromone	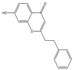	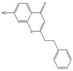
43	FTPECs	2-[2-(3-hydroxy-4-methoxyphenyl)ethyl]chromone		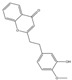
51	FTPECs	2-[2-(2-hydroxyphenyl)ethyl]chromone		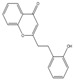
56	FTPECs	6-hydroxy-2-[2-(3-methoxyphenyl)ethyl]chromone		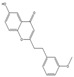
58	FTPECs	6-hydroxy-2-(2-phenylethyl)chromone	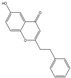	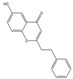
60	FTPECs	6-methoxy-2-[2-(3-methoxyphenyl)ethyl]chromone		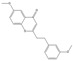
75	FTPECs	2-[2-(4-methoxyphenyl)ethyl]chromone	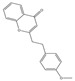	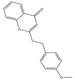
78	FTPECs	2-(2-phenylethyl)chromone	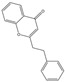	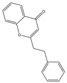

**Table 3:** The log Po/w and TPSA of the 21 brain-penetrating chromone components.

No.	Type	Component Name	Log Po/w	TPSA
6	THPECs	Agarotetrol	0.27	111.13
7	THPECs	4′-methoxyagarotetrol	0.26	120.36
10	THPECs	Agarotetrol (isomer 1)	0.27	111.13
18	THPECs	8-chloro-5,6,7-trihydroxy-2-(4-methoxyphenethyl)-5,6,7,8-tetrahydrochromene	1.09	91.44
19	THPECs	8-chloro-5,6,7-trihydroxy-2-(2-phenylethyl)-5,6,7,8- tetrahydrochromon	1.16	90.90
21	FTPECs	6-hydroxy-2-[2-(4-hydroxyphenyl)ethyl]chromone	2.89	70.67
24	FTPECs	6-hydroxy-2-[2-(4-hydroxyphenyl)ethyl]chromone	2.89	70.67
26	FTPECs	6-hydroxy-2-[2-(3-hydroxy-4-methoxylphenyl)ethyl]chromone	2.77	79.9
28	FTPECs	6-hydroxy-2-[2-(3-methoxyl-4-hydroxyphenyl)ethyl] chromone	2.76	79.9
30	FTPECs	6-methoxy-2-[2-(3-hydroxy-4-methoxyphenyl) ethyl]chromone	3.17	68.9
33	FTPECs	2-[2-(3-hydroxyphenyl)ethyl]chromone	3.22	50.44
39	FTPECs	2-[2-(4-hydroxyphenyl)ethyl]chromone	3.21	50.44
40	FTPECs	2-[2-(3-methoxy-4-hydroxyphenyl)ethyl]chromone	3.16	59.67
41	FTPECs	7-hydroxy-2-(2-phenylethyl)chromone	3.29	50.44
43	FTPECs	2-[2-(3-hydroxy-4-methoxyphenyl)ethyl]chromone	3.17	59.67
51	FTPECs	2-[2-(2-hydroxyphenyl)ethyl]chromone	3.13	50.44
56	FTPECs	6-hydroxy-2-[2-(3-methoxyphenyl)ethyl]chromone	3.13	59.67
58	FTPECs	6-hydroxy-2-(2-phenylethyl)chromone	3.26	50.44
60	FTPECs	6-methoxy-2-[2-(3-methoxyphenyl)ethyl]chromone	3.54	48.67
75	FTPECs	2-[2-(4-methoxyphenyl)ethyl]chromone	3.61	39.44
78	FTPECs	2-(2-phenylethyl)chromone	3.63	30.21

**Table 4:** The 10 compounds detected in the medicated serum of agarwood.

Number	Name
TH-1	Agarotetrol
TH-2	4′-methoxyagarotetrol
TH-3	Isoagarotetrol
TH-4	4’-methoxyisoagarotetrol
TH-5	6,7-dihydroxy-2-(2-phenylethyl)-5,6,7,8-tetrahydrochromone
TH-6	(5S,6S,7S,8R)-8-chloro-5,6,7-trihydroxy-2-(2-phenylethyl)-5,6,7,8-tetrahydrochromen-4-one
FT-1	2-(2-phenylethyl)chromone
FT-2	6-hydroxy-2-(2-phenylethyl)chromone
FT-3	2-[2-(4’-methoxyphenyl)ethyl]chromone
FT-4	6-methoxy-2-[2-(3’-methoxyphenyl)ethyl]chromone

## Data Availability

All data generated or analyzed during this study are included in this published article and its Supplementary Materials files.

## Supplementary Materials

The following supporting information can be downloaded at https://www.mdpi.com/article/10.3390/ph18040510/s1, Table S1: Analytical results of the chromone components of two types of agarwood detected using UPLC-MS; Table S2: Analysis of metabolites in serum containing two types of agarwood; File S1: Sample information of two types of agarwood.

## Abbreviations

The following abbreviations are used in this manuscript:

OAE	ordinary agarwood ethanol extract
QNE	Qi-Nan agarwood ethanol extract
OAS	drug-containing serum of rats in ordinary agarwood ethanol extract
QNS	drug-containing serum of rats in Qi-Nan agarwood ethanol extract
OAB	brain tissue of rats in ordinary agarwood ethanol extract
QNB	brain tissue of rats in Qi-Nan agarwood ethanol extract
Herb-Q	herb molecular quantification
UPLC-MS	ultra performance liquid chromatography–mass spectrometry
DESI-MSI	desorption electrospray ionization–imaging mass spectrometry
FTPECs	Flindersia-type 2-(2-phenylethyl)chromones
THPECs	5,6,7,8-tetrahydro-2-(2-phenylethyl)chromones
CCK-8	Cell Counting Kit-8
LPS	lipopolysaccharide
DEX	dexamethasone
DMSO	dimethylsulfoxide
IL-6	Interleukin 6
TNF-α	tumor necrosis factor alpha
log Po/w	N-octanol/water partition coefficient
TPSA	topological polar surface area
