# Supplementary material for: Comparison of the Blood–Brain Barrier Penetration Ability and Anti-Neuroinflammatory Activity of Chromones in Two Types of Agarwood
Source: Pharmaceuticals (Basel). 2025 Mar 31;18(4):510. doi: 10.3390/ph18040510 (PMC12030442; doi:10.3390/ph18040510)
Supplement: Supplementary file 1 [file pharmaceuticals-18-00510-s001.zip › File S1.pdf]

The 12 Ordinary Agarwood and 12 Qi-Nan Agarwood samples were obtained from two main producing areas, namely, Guangdong (20°13′–25°31′ N, 109°39′–117°19′ E) and Hainan (3°58′–20°20′ N, 108°37′–117°50′ E) Province, China, to exclude the influence of other factors, such as growth conditions. The details of the 24 agarwood samples are shown in Table S1. Besides, Ordinary Agarwood samples were authenticated by Professor Zhijie Zhang of the Institute of Chinese Materia Medica, China Academy of Chinese Medical Sciences. Qi-Nan Agarwood samples were authenticated by Shan Sun, President of the Beijing Agarwood Association.

12 batches of Ordinary Agarwood were tested according to the regulations of "China Pharmacopoeia". All the test results of the 12 Ordinary Agarwood meet the requirements of "China Pharmacopoeia". Therefore, according to the ethanol extract content of Ordinary Agarwood, we choose Ordinary Agarwood with higher ethanol extract content (Figure S2).

Through the determination of the fingerprint of 12 batches of Qi-Nan Agarwood, it is concluded that the similarity of the fingerprint of 12 batches of Qi-Nan Agarwood(Figure 1). Therefore, according to the content of ethanol extract, we choose Qi-Nan Agarwood with higher ethanol extract content (Figure S2).

Table S1. The details of the 24 agarwood samples

Num.	Type	Origin	Num.	Type	Origin
S1	Qi-Nan Agarwood	Guangdong, China	OA1	Ordinary Agarwood	HaiNan, China
S2	Qi-Nan Agarwood	Guangdong, China	OA2	Ordinary Agarwood	HaiNan, China
S3	Qi-Nan Agarwood	Guangdong, China	OA3	Ordinary Agarwood	Hong Kong, China
S4	Qi-Nan Agarwood	Guangdong, China	OA4	Ordinary Agarwood	Guangdong, China
S5	Qi-Nan Agarwood	Guangdong, China	OA5	Ordinary Agarwood	Guangdong, China
S6	Qi-Nan Agarwood	Guangdong, China	OA6	Ordinary Agarwood	HaiNan, China
S7	Qi-Nan Agarwood	Guangdong, China	OA7	Ordinary Agarwood	HaiNan, China
S8	Qi-Nan Agarwood	Guangdong, China	OA8	Ordinary Agarwood	HaiNan, China
S9	Qi-Nan Agarwood	Guangdong, China	OA9	Ordinary Agarwood	HaiNan, China
S10	Qi-Nan Agarwood	Guangdong, China	OA10	Ordinary Agarwood	Guangdong, China
S11	Qi-Nan Agarwood	Guangdong, China	OA11	Ordinary Agarwood	Guangdong, China
S12	Qi-Nan Agarwood	HaiNan, China	OA12	Ordinary Agarwood	Guangdong, China

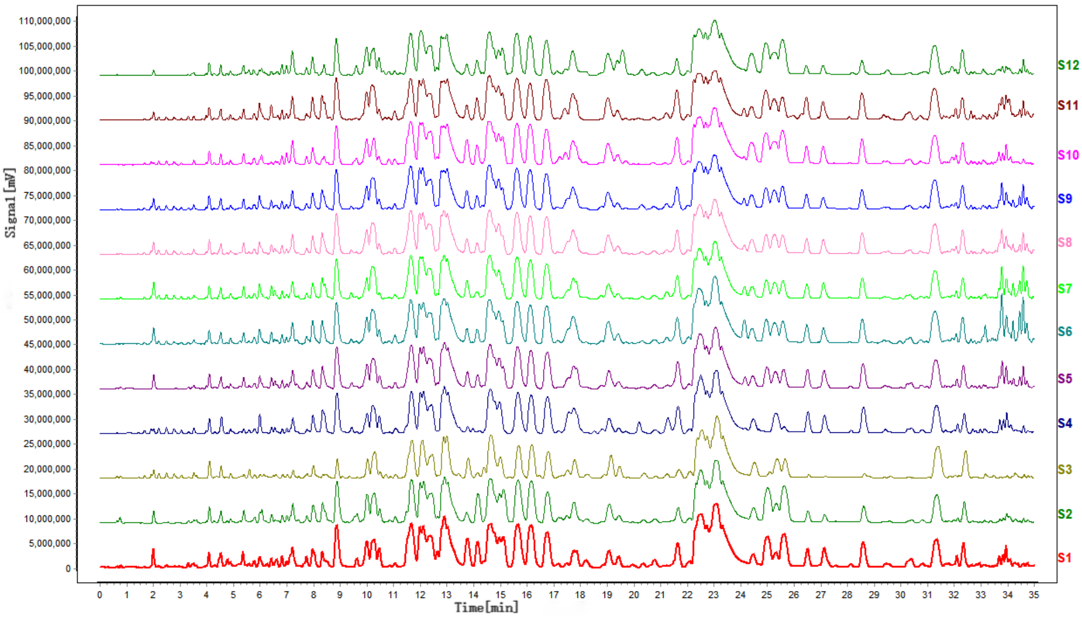

Figure S1. Superimposed fingerprints of 12 batches of Qi-Nan Agarwood.

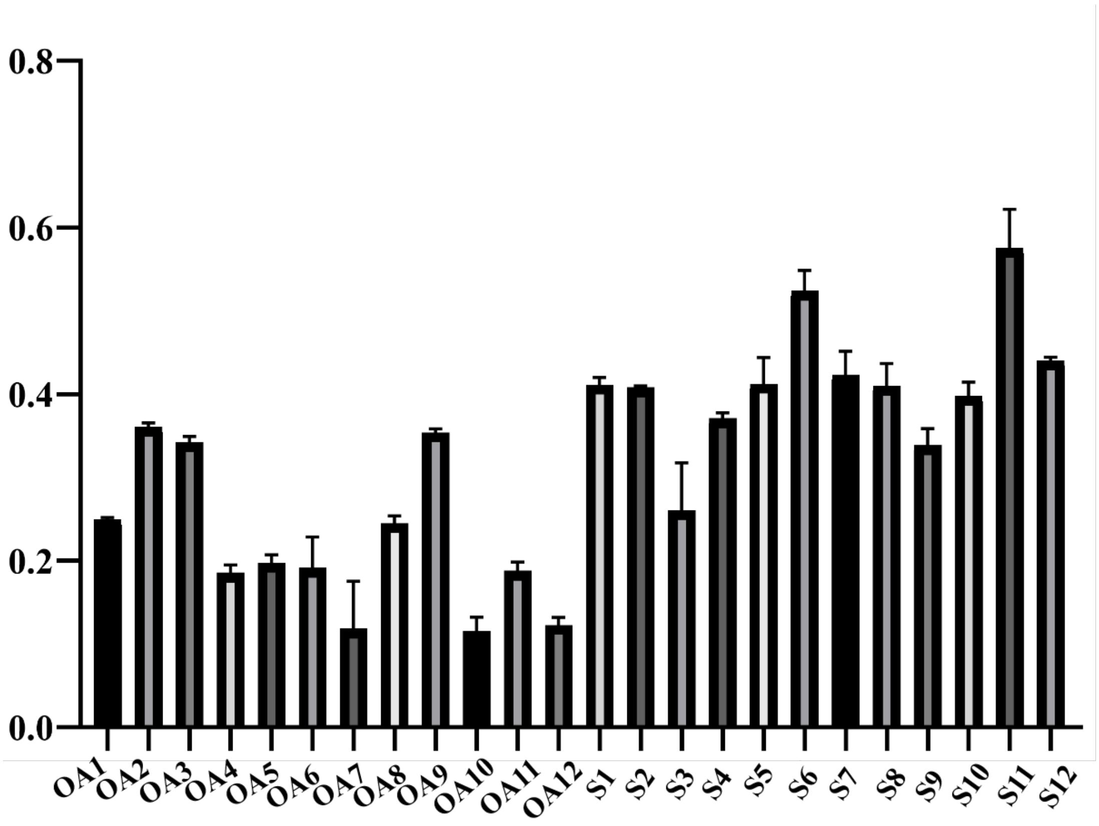

Figure S2. Determination results of alcohol extracts from Ordinary Agarwood and Qi-Nan Agarwood (OA1-12 is Ordinary Agarwood and S1-12 is Qi-Nan Agarwood)
